# Effect of Feeding Status on Adjuvant Arthritis Severity, Cachexia, and Insulin Sensitivity in Male Lewis Rats

**DOI:** 10.1155/2010/398026

**Published:** 2010-09-30

**Authors:** Andrea Stofkova, Blanka Zelezna, Marianna Romzova, Olga Ulicna, Alexander Kiss, Martina Skurlova, Jana Jurcovicova

**Affiliations:** ^1^Department of Normal, Pathological, and Clinical Physiology, Third Faculty of Medicine, Charles University in Prague, Ke Karlovu 4, 120 00 Prague, Czech Republic; ^2^Institute of Organic Chemistry and Biochemistry, Academy of Sciences of the Czech Republic, 116610 Prague, Czech Republic; ^3^Institute of Biotechnology, Academy of Sciences of the Czech Republic, 14220 Prague, Czech Republic; ^4^Third Department of Internal Medicine, Faculty of Medicine, Comenius University, 81108 Bratislava, Slovakia; ^5^Institute of Experimental Endocrinology, Slovak Academy of Sciences, 83306 Bratislava, Slovakia

## Abstract

We studied the effect of food restriction, overfeeding, and normofeeding on cachexia, inflammatory and metabolic parameters, and insulin sensitivity in chronic adjuvant arthritis (AA) in rats. Food restriction during AA increased circulating ghrelin, corticosterone, decreased leptin, and ameliorated arthrogram score and systemic inflammation compared to normofeeding. Overfeeding worsened arthrogram score and systemic inflammation, and led to lipid accumulation in the liver, but not to alterations of adipokine and ghrelin plasma levels relative to normofeeding. Independently of feeding status, AA induced cachexia, in which modulation of mRNA expressions for appetite-regulating neuropeptides (NPY, AgRP, POMC, CART) in the arcuate nucleus (ARC) does not play a primary role. The overexpression of IL-1*β* mRNA in the ARC suggests its role in the mechanisms of impaired energy balance during AA under all feeding conditions. Normal HOMA index in all arthritic groups does not indicate the development of insulin resistance by feeding interventions in these rats.

## 1. Introduction

The world wide growing incidence of obesity has triggered increased research into the adverse health consequences of this condition. Particular attention has been paid to white adipose tissue- (WAT-) derived adipokines. At present, there is a large body of evidence that chronic subclinical inflammation associated with changes in endocrine functions of adipose tissue leads to metabolic disturbances including development of insulin resistance, diabetes, and cardiovascular disease [1]. However, an important question still remains. Whether increased levels of systemic inflammation during obesity can also predispose susceptible individuals to the development of autoimmune inflammatory diseases such as rheumatoid arthritis (RA).

The relationship between obesity and RA is difficult to settle as chronic RA brings about cachexia, and there is little documentation on body composition before the onset of the disease. The literature describing the link between obesity and RA failed to show differences in prevalence of RA between obese and nonobese patients [2]. It has to be noted that severe rheumatoid disease is associated with inflammatory cachexia characterized by the loss of lean body tissue that is often compensated for by gain in body fat. Thus about 85% of RA patients have a normal body mass index (BMI) [3]. Recently, it has been described that higher BMI was associated with increased leptin levels and a less severe radiographic joint damage of small joints in patients with RA [4, 5]. In contrast, leptin has been shown to positively correlate with the disease activity score in 28 joints (DAS28), and the IL-6, and CRP levels [5]. Interestingly, reduction of leptin levels in patients with RA due to starving was accompanied by decreased CD4+ lymphocyte activation and increased IL-4 production, which resulted in attenuation of disease activity [6]. Under experimental conditions, in rodent models of RA, chronic arthritis was less severe in food restricted rats [7], or in *ob/ob* mice with leptin deficiency [8].

Leptin is an adipokine with proinflammatory nature that can promote immune system reactivity and autoimmunity [9–11]. It positively correlates with BMI and suppresses appetite through regulation of specific hypothalamic neuropeptides: pro-opiomelanocortin (POMC), cocaine- and amphetamine-regulated transcript (CART), neuropeptide Y (NPY), and agouti-related peptide (AgRP) [12]. Oppositely to leptin, gastric hormone ghrelin negatively correlates with BMI, stimulates appetite at the hypothalamic level [13], and inhibits leptin-induced expression of pro-inflammatory cytokines [14]. Another adipokine, adiponectin, acts as a protective factor in some inflammation connected pathologies such as atherosclerosis, cardiovascular disease, insulin resistance, and type 2 diabetes mellitus. Both proinflammatory and antiinflammatory effects of adiponectin have been reported with regard to joint inflammation [10]. Visfatin is an adipokine known for its insulin-mimicking/-sensitizing effects, pro-inflammatory properties, as well as for its activation by inflammation [15]. 

Because numerous studies have indicated that adipokines can play a role in joint degenerative diseases including inflammatory arthritis [16], it can be hypothesized that obesity-induced changes in adipokine levels towards inflammatory phenotype may worsen the outcome of arthritis. Conversely, fasting associated with body fat loss, and increased ghrelin levels may lead to antiinflammatory phenotype, and to milder symptoms of arthritis. Therefore, in this study we investigated the effect of feeding status (overfeeding, normofeeding, and food restriction) on chronic arthritis severity, arthritis-associated cachexia, and metabolic responses. For overfeeding we have used a model of early-life overnutrition induced by reduced litter size and followed by high-fat diet consumption. This model produces accumulation of fat mass, hyperleptinemia, and hypoadiponectinemia by the age of 8 weeks [17]. The model of 40% food restriction was used on the basis of our previous work, where we observed the improvement of arthritis, but adipokines were not examined in that study [7]. In this study we are describing interventions of leptin, ghrelin, adiponectin, and visfatin with inflammation, hepatic lipid accumulation, insulin sensitivity, and hypothalamic orexigenic and anorexigenic peptide expressions in high-fat diet fed, food restricted, and normally fed arthritic rats.

## 2. Materials and Methods

### 2.1. Animals and Experimental Design

Male 21-days old Lewis rats, after birth adjusted to litter sizes of 4 (small litter = SL) or 8 (normal litter = NL) pups with their nursing females were obtained from Charles River (Germany). The litters were kept under 12-h light/dark cycle (light on from 6 : 00 a.m. to 6 : 00 p.m.) with controlled humidity and temperature on standard pellet diet in an animal room of the Department of Normal, Pathological and Clinical Physiology, Third Faculty of Medicine, Charles University in Prague. On day 23 of life the pups were separated from their nursing females, and were housed 4 per cage. The NL pups were given a standard rodent diet containing 10% calories as fat (ST1, Velaz, Czech Republic), and the SL pups were given a high-fat diet (D12451; Ssniff, Germany) containing 45% calories as fat [39.5% lard (containing 39% saturated fat, 56% unsaturated fat, and 0.95% cholesterol) and 5.5% soybean oil (containing 16% saturated fat and 81% unsaturated fat)]. Both groups had free access to water and respective diets. 

At the age of 8 weeks the rats were divided into following 6 groups according to dietary regime and AA induction: normally fed controls from NL (CN; *n* = 8); normally fed arthritic rats from NL (AA-N; *n* = 8); high-fat diet fed controls from SL (SHF; *n* = 8); high-fat diet fed arthritic rats from SL (AA-SHF; *n* = 8); food-restricted controls from NL (FR; *n* = 8); food-restricted arthritic rats from NL (AA-FR; *n* = 8). FR groups were given 60% of the standard diet consumed by their normally fed counterparts on the previous day. Diets were given regularly between 8 a.m. and 9 a.m., and food consumption per cage and body mass were measured daily at the same time of day. AA was induced to rats by a single subcutaneous injection of 100 *μ*L of complete Freund's adjuvant (cFA) 2 cm from the base of the tail as described previously in [18]. These rats had cage feeder assembled for easy food access. 

The rats were decapitated on day 18 of AA after a 12 h fast. Trunk blood was collected into tubes with EDTA, centrifuged and plasma was stored at –70°C. Mesenteric and epididymal adipose tissues were dissected and weighed. Liver and brain was dissected, snapfrozen in liquid nitrogen, and stored at –70°C until analyzed. All the organs were collected in a native form without perfusion because in integrated organism the contribution of factor/cell present in the blood or in the individual organ is not distinguishable. The animals were treated in accordance with the national law of the Czech Republic on the use of laboratory animals no. 246/1992 (fully compatible with European Community Council directives 86/609/EEC) based on the project approved by the Committee for Protection of Experimental Animals at the Third Faculty of Medicine, Prague.

### 2.2. Arthrogram Score

Paw swelling was measured volumetrically on days 12, 15, and 18 after AA induction. The periarticular erythema and swelling of each paw was evaluated by scoring from 1 to 5. The severity of arthritis was quantified by the sum of the scores for the paws, with the maximum possible scores of 20 per rat (Svik, personal communication).

### 2.3. Hypothalamic Arcuate Nucleus Isolation

The hypothalamic arcuate nucleus (ARC) was isolated from the frozen brains by a punching technique [19]. 

### 2.4. Plasma Determinations and Homa Index

All parameters were measured after a 12 h fast. Levels of plasma leptin, adiponectin, visfatin, insulin, and total ghrelin were determined by specific rat RIA kits from Linco Research (St. Charles, MO). Corticosterone plasma levels were determined by the rat and mouse corticosterone high sensitivity EIA kit (IDS, Boldon, UK). Plasma CRP levels were measured by the commercial rat ELISA kit from ICL (Newberg, OR). The basal level of glucose in plasma was measured by the autoanalyser Hitachi 911 (Hitachi, Japan). Homeostasis model assessment (HOMA) index was calculated using the following formula: fasting plasma insulin (*μ*U/mL) × fasting plasma glucose (mmol/L)/22.5.

### 2.5. Triglyceride and Cholesterol Determinations in the Liver

Triglyceride concentrations in the liver were determined by the modified method of Jover [20]. Liver tissue (100 mg) was homogenized and extracted in chloroform/methanol (2 : 1). The interfering phospholipids were removed by absorption from the liver extract on silica gel. Purified extracts were evaporated and triglycerides hydrolyzed with potassium hydroxide. Released fatty acids were removed by extraction into heptane. Finally, the released glycerol was oxidized by periodic acid, and after the reaction with phenylhydrazine, a colored complex was measured spectrophotometrically at 530 nm. Cholesterol was determined by the modified method of Abel et al. [21]. Liver tissue (100 mg) was homogenized in chloroform/methanol (1 : 1). After lipid extraction, the Lieberman-Burchard colorimetric assay was used for the detection of cholesterol. Cholesterol concentrations were determined spectrophotometrically at 650 nm.

### 2.6. RNA Preparation and Quantitative TaqMan PCR Procedure in the Liver

Liver tissue was homogenized using QIAshredder (Qiagen, Valencia, CA). Total RNA was isolated using RNeasy Mini Kit (Qiagen, Valencia, CA). The RNA samples were reversely transcribed to cDNA using SuperScript III RT enzyme (Invitrogen, Carlsbad, CA). The quantitative PCR reaction was performed using TaqMan Assays (Applied Biosystems, Foster City, CA). Fluorogenic probe for control GAPDH gene was labeled with the VIC reporter dye. Probes for both target genes CRP and IL-1*β* were labeled with the FAM reporter dye. The reactions contained TaqMan Universal Master Mix (Applied Biosystems, Foster City, CA). Samples were run in triplicates. The thermal cycling proceeded according to manufacturer's protocol: 40 cycles of 95°C for 15 s and 60°C for 1 min with one initial setup step 10 min 95°C. Data were collected using CFX96 Real-time system (Bio-Rad, Hercules, CA). Input RNA amounts were calculated with a multiple comparative method for the mRNAs of interest and GAPDH. Statistical analysis was performed using Genex Ver. 5 software (MultiD Analyses AB, Goteborg, Sweden). 

### 2.7. RNA Preparation and Quantitative TaqMan PCR Procedure in the ARC

Poly(A)RNA was isolated from the ARC using Chemagic mRNA Direct Kit (Chemagen Biopolymer-Technologie, Beasweiler, Germany). The signal was reversely transcribed to cDNA by a reaction containing Omniscript RT Kit (Qiagen, Valencia, CA) components (RT buffer, RNase free water, dNTP mix, Omniscript Reverse Transcriptase), RNase inhibitor (Takara, Shiga, Japan), and pd(N)_6_ random hexamer primers (Amersham Biosciences, Piscataway, NJ). The expressions of NPY, AgRP, POMC, CART, and IL-1*β* were quantified using ABI Prism 7000 Sequence Detector and System Software (Applied Biosystems, Foster City, CA). The reactions were performed with TaqMan gene expression products (Applied Biosystems, Foster City, CA) as detailed in our previous study [19].

### 2.8. Statistical Analysis

For the statistical analysis it was used Welch version of two-sample *t*-test with unequal variances. Bonferroni correction was applied to treat the multiple comparisons. Adjusted *P* value <.05 was considered statistically significant. Data are presented as means ± SEM for each group.

## 3. Results

### 3.1. Body Mass, Food Intake, and Arthrogram Score

**Figure 1:**
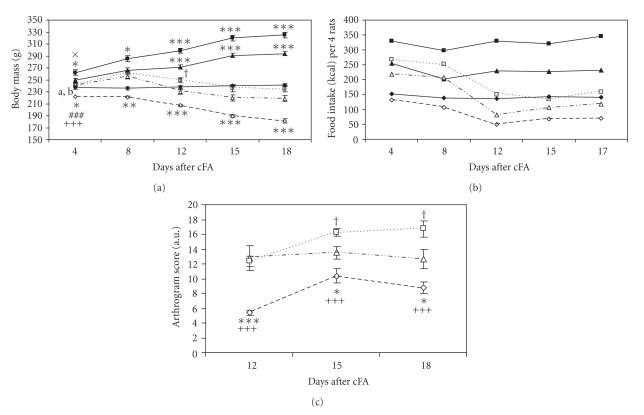
Body mass (a) and food intake (b) in control rats on standard diet (CN  = ▲), high-fat diet (SHF  =  ■), and food restriction (FR  = ♦), and in arthritic rats on standard diet (AA-N  =  ∆), high-fat diet (AA-SHF  =  □), and food restriction (AA-FR =  *◊*) in selected days after cFA inoculation. Values for food intake are presented as means of 2 cages (4 rats per cage). Statistical significance between the body mass values in arthritic rats and their corresponding controls on the same dietary regime: *P < .05, **P < .01, ***P < .001. Significant difference between AA-N and AA-SHF: ^†^ *P* < .05. First day of significant differences: CN versus SHF ^×^ *P* < .05, FR versus CN ^a^ *P* < .05, FR versus SHF ^b^ *P* < .001, AA-FR versus AA-N ^###^ *P* < .001, and AA-FR versus AA-SHF ^+++^ *P* < .001. Effects of dietary interventions on the severity of arthritis expressed by arthrogram score (c). Significant difference between AA-FR and AA-N: *P < .05, ***P < .001; significant difference between AA-FR and AA-SHF: ^+++^ *P* < .001; significant difference between AA-SHF and AA-N: ^†^ *P* < .05.

Chronic inflammatory arthritis was associated with severe weight loss in all groups. It started 10 days after cFA injection in normally fed arthritic rats and continued throughout the experiment. However, weight loss of food-restricted arthritic and arthritic rats on high-fat diet was not visible until the day 4 of AA. In spite of the fact that control rats on high-fat diet gained weight in considerably higher rate than normally fed control rats from the 9th week of life onwards, arthritic rats on both diets had similar body mass during the whole course of the disease. However, we observed significantly higher amounts of mesenteric fat (AA-SHF 358.00 ± 67.4 mg versus AA-N 174.29 ± 32.7 mg, *P  *< .001) and epididymal fat (AA-SHF 542.88 ± 58.3 mg versus AA-N 360.13 ± 18.3 mg, *P* < .001) in AA rats on high-fat diet than in normally fed AA rats. As expected, control and arthritic food-restricted rats displayed decreased body mass values from day 4 to 18 after the injection of cFA (Figure 1(a)).

In the arthritic groups as well as in the control groups, the values of calorie intake per cage reflected the values of body mass (Figure 1(b)). The clinical signs such as redness, and swelling of paws appeared from day 12 of AA onwards in all arthritic groups. Significant reduction in arthrogram score was observed in food-restricted arthritic rats, and conversely, arthritic rats on high-fat diet had increased values of arthrogram score compared to the other arthritic groups (Figure 1(c)).

### 3.2. Corticosterone, CRP, and IL-1*β*

**Figure 2:**
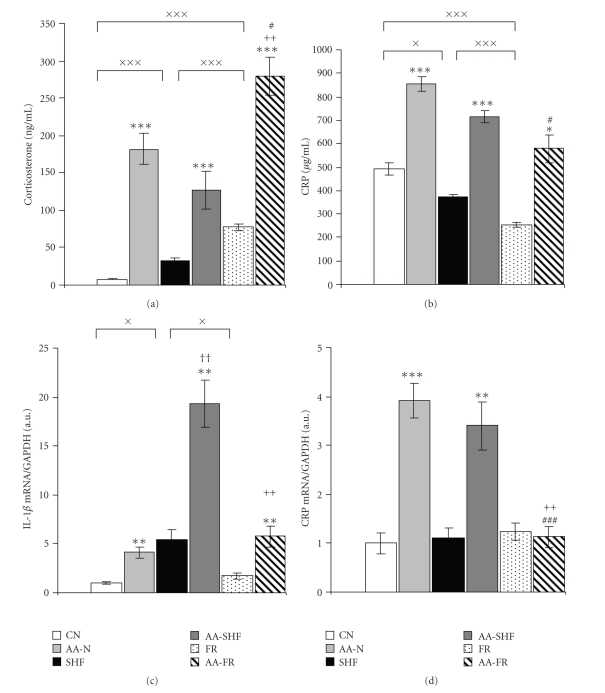
Plasma corticosterone (a), plasma CRP (b), IL-1*β* mRNA expression (c), and CRP mRNA expression (d) in the liver of healthy control (CN, SHF, FR) and arthritic rats (AA-N, AA-SHF, AA-FR) measured on day 18 after cFA inoculation. Results of mRNA expressions are expressed in arbitrary units as the ratio of given mRNA to GAPDH. Significant differences between healthy controls on different dietary regime: ^×^ *P* < .05, ^×××^ *P* < .001. Statistical significances: control rats versus arthritic rats on the same dietary regime *P < .05, **P < .01, ***P < .001; AA-FR versus AA-N ^#^ *P* < .05, ^###^ *P* < .001; AA-FR versus AA-SHF ^++^ *P* < .01; AA-SHF versus AA-N ^††^ *P* < .01. For abbreviations see Figure 1.

Plasma corticosterone levels were affected by arthritis as well as by the dietary interventions. All arthritic groups had significantly elevated corticosterone compared to their respective controls, moreover corticosterone level of food-restricted arthritic rats was significantly more pronounced than in the other arthritic rats. There were also significant changes in corticosterone levels among the control groups, showing mild increase in control rats on high-fat diet compared to control rats on normal diet, and a very pronounced increase in food-restricted rats compared to normally fed and high-fat diet fed rats (Figure 2(a)). The levels of plasma CRP as well as the mRNA expressions of CRP in the liver were significantly elevated in normally fed and high-fat diet fed arthritic rats compared to their respective controls. The activation of plasma CRP in the arthritic rats on food-restriction was significantly less pronounced than in the other arthritic groups, and moreover, its hepatic mRNA expression was not enhanced at all. In the control rats food restriction reduced plasma CRP levels compared to the other 2 control groups, and a slight but significant decrease of CRP plasma levels occurred also in control rats on high-fat diet compared to normally fed control rats (Figures 2(b) and 2(d)). Chronic inflammatory arthritis caused clear-cut increase in IL-1*β* mRNA expression in the liver in all dietary modulated groups with several times higher values in arthritic rats on high-fat diet over the normally fed or foodrestricted arthritic rats. Similarly, control rats on high-fat diet displayed increased IL-1*β* mRNA expression in the liver relative to normally fed or food-restricted control rats (Figure 2(c)). The IL-1*β* expression represents a pool originating from liver cells and also from circulating white blood cells accumulated in the liver, since we used native liver without perfusion.

### 3.3. Adipokines and Ghrelin

**Figure 3:**
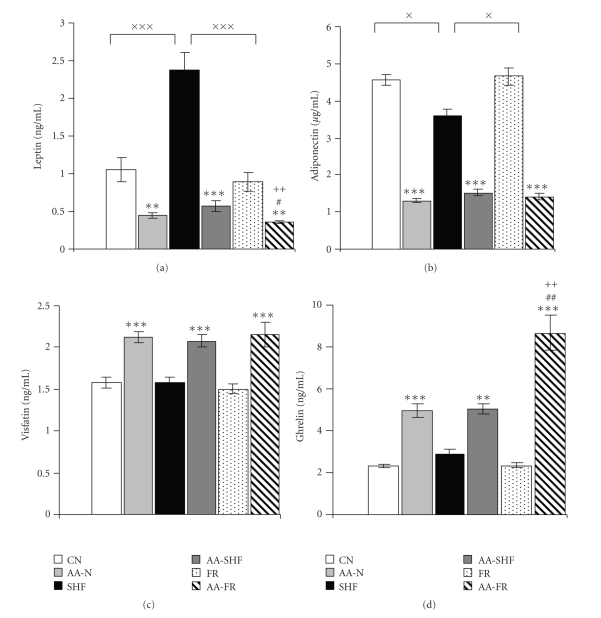
Plasma leptin (a), adiponectin (b), visfatin (c) and (d) ghrelin levels in healthy control (CN, SHF, FR) and arthritic rats (AA-N, AA-SHF, AA-FR) on day 18 after cFA inoculation. Significant differences between healthy controls on different dietary regime: ^×^ *P* < .05, ^×××^ *P* < .001. Significant differences between control rats and arthritic rats on the same dietary regime: **P < .01, ***P < .001. Significant differences between dietary modulated arthritic groups: ^#^ *P* < .05, ^##^ *P* < .01 (AA-FR versus AA-N); ^++^ *P* < .01 (AA-FR versus AA-SHF). For abbreviations see Figure 1.

Plasma leptin and adiponectin levels were significantly decreased in all arthritic groups comparing to their corresponding controls. Overfeeding resulted in the increase of leptin and decrease of adiponectin levels compared to normofeeding or food restriction only in control, but not in arthritic rats (Figures 3(a) and 3(b)). In addition, food restriction during arthritis resulted in significantly lower levels of leptin compared to the other dietary modulated arthritic rats (Figure 3(a)). Plasma visfatin levels increased equally in all arthritic groups of rats comparing to their respective controls. There were no differences in visfatin values with respect to the dietary interventions in arthritic rats (Figure 3(c)). All groups of arthritic rats had higher plasma ghrelin levels than their corresponding controls. The food-restricted arthritic rats showed significantly enhanced ghrelin levels compared to the other arthritic rats (Figure 3(d)).

### 3.4. Neuropeptide and IL-1*β* mRNA Expressions in the ARC

**Figure 4:**
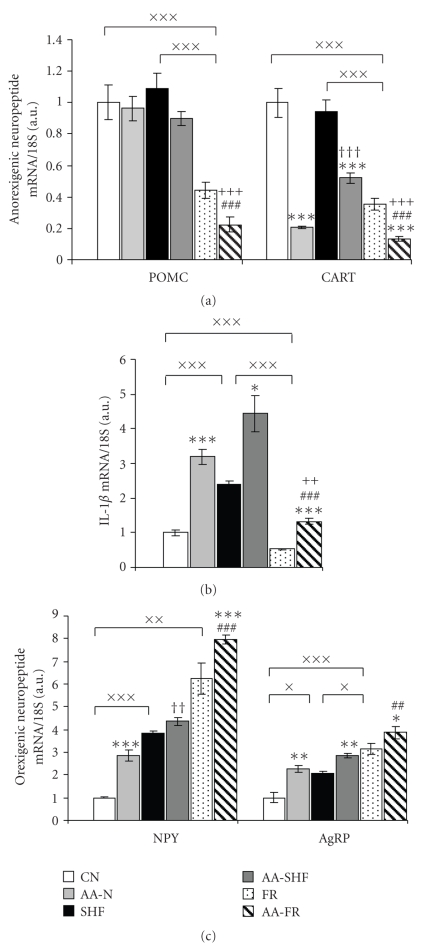
Anorexigenic (a) and orexigenic (c) neuropeptide and IL-1*β* (b) mRNA expressions in the ARC in healthy control (CN, SHF, FR) and arthritic rats (AA-N, AA-SHF, AA-FR) on day 18 after cFA inoculation. Results are expressed in arbitrary units as the ratio of given mRNA to 18S rRNA. Significant differences between healthy controls on different dietary regime: ^×^ *P* < .05, ^××^ *P* < .01, ^×××^ *P* < .001. Significant differences between control rats and arthritic rats on the same dietary regime: *P < .05, **P < .01, ***P < .001. Significant differences between arthritic groups: ^##^ *P* < .01, ^###^ *P* < .001 (AA-FR versus AA-N); ^++^ *P* < .01, ^+++^ *P* < .001 (AA-FR versus AA-SHF); ^††^ *P* < .01, ^†††^ *P* < .001 (AA-SHF versus AA-N). For abbreviations see Figure 1.

The mRNA expressions of the anorexigenic POMC in the ARC did not differ between arthritic rats and their corresponding controls. However, the mRNA expressions of the anorexigenic CART were significantly lower in all arthritic rats comparing to their controls. It is important to note that food restriction resulted in decreased values of both POMC and CART mRNA expressions compared to the other dietary regimes with or without arthritis. Overfeeding in arthritic rats increased mRNA expression of CART compared to normofeeding in arthritic rats (Figure 4(a)). The mRNA expression of IL-1*β* in the ARC was significantly increased by chronic inflammatory arthritis in all dietary modulated rats. Food restriction led to the lower IL-1*β* mRNA expressions in both arthritic and control rats than in the other dietary modulated arthritic and control rats. In contrast, overfeeding led to the enhanced IL-1*β* mRNA expression compared to normofeeding only in controls (Figure 4(b)). Evaluation of the mRNA expression for the orexigenic NPY in the ARC showed its upregulation during arthritis. Moreover, in control groups both food restriction and overfeeding caused increased NPY mRNA in the ARC compared to normofeeding. Therefore, no significant differences could be revealed between control and arthritic rats on high-fat diet, or between food-restricted control and arthritic rats. However, both food-restricted and high-fat diet fed arthritic rats showed enhanced NPY mRNA expressions over normally fed arthritic rats, and food-restricted arthritic rats showed enhanced NPY mRNA compared to arthritic rats on high-fat diet. Chronic inflammatory arthritis significantly enhanced mRNA expression for another orexigenic peptide, AgRP in all dietary modulated groups. This effect was more pronounced in food-restricted arthritic rats compared to normally fed arthritic rats. Similarly as NPY mRNA, AgRP mRNA expression was increased in both food-restricted and high-fat diet fed control groups. Plus, the values of AgRP mRNA in food restricted controls were more pronounced than those in controls on high-fat diet (Figure 4(c)).

### 3.5. Parameters of Metabolism

**Figure 5:**
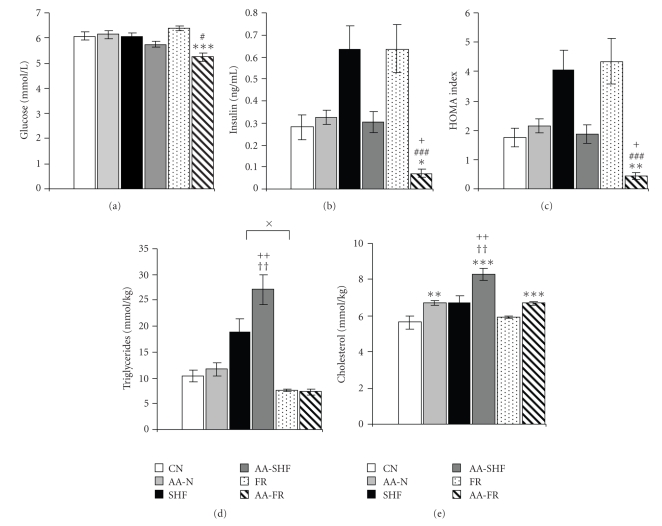
Basal plasma levels of glucose (a) and insulin (b), HOMA index (c), and liver concentrations of triglycerides (d) and cholesterol (e) in healthy controls (CN, SHF, FR) and arthritic rats (AA-N, AA-SHF, AA-FR) on day 18 after cFA inoculation. Results are expressed as means ± SEM. Significant difference between healthy controls on different dietary regime: ^×^ *P* < .05. Significant differences between control rats and arthritic rats on the same dietary regime: *P < .05, **P < .01, ***P < .001. Other statistical differences: AA-FR versus AA-N ^#^ *P* < .05, ^###^ *P* < .001; AA-FR versus AA-SHF ^+^ *P* < .05, ^++^ *P* < .01; AA-SHF versus AA-N ^††^ *P* < .01. For abbreviations see Figure 1.

Food restriction during arthritis led to a reduction of basal glucose, and insulin plasma levels, and consequently to a reduction of HOMA index compared to food restriction in controls or to the other dietary regimes during arthritis (Figures 5(a), 5(b), and 5(c)). Overfeeding resulted in enhanced triglyceride and cholesterol concentrations in the liver of arthritic rats compared to those on the other dietary regimes (Figures 5(d) and 5(e)). In addition, control rats on high-fat diet had also higher triglyceride concentrations than control food-restricted rats (Figure 5(d)). Interestingly, arthritis in all dietary regimes led to the increased cholesterol levels (Figure 5(e)).

## 4. Discussion

This study shows that feeding status modulates the disease severity, mRNA levels of IL-1*β* and of the key neuropeptides regulating appetite in the ARC, as well as metabolic responses in chronic adjuvant arthritis. 

### 4.1. Feeding Status and AA Severity

Our study confirmed previous observations that AA under the condition of normofeeding decreased circulating levels of leptin [18, 22, 23], and adiponectin [19, 23, 24], and increased circulating levels of visfatin [25], ghrelin [19, 26], and corticosterone [7, 18, 19, 27]. Regarding the severity of arthritis, the most important finding of this investigation is the reduced arthrogram score associated with a profound decrease of circulating leptin and a marked increase of circulating ghrelin and corticosterone levels due to the food restriction. This finding is in a good agreement with the known immunomodulatory functions of the respective hormones. Downregulation of leptin or its deficiency has been shown to prevent Th1-mediated autoimmune diseases such as RA [6], autoimmune arthritis [8], and encephalomyelitis in mice [28]. Administration of ghrelin or ghrelin agonists stimulated antiinflammatory responses in various autoimmune inflammatory diseases including AA in rats [22, 29–31]. Glucocorticoids are known to regulate Th1/Th2 lymphocyte balance towards Th2 pattern by suppressing Th1 cytokine production [32]. Consistently with the improvement of arthrogram score in food-restricted arthritic rats, we observed an attenuation of inflammatory parameters such as a decrease of CRP plasma levels, and of CRP and IL-1*β* mRNA expressions in the liver. This suggests that the food restriction-induced inhibition of leptin, activation of ghrelin, and corticosterone contributed to the attenuation of the disease. 

In this study, overfeeding led to a worsened arthrogram score along with stronger inflammatory response manifested by a higher IL-1*β* mRNA expression in the liver. However, plasma leptin and adiponectin levels in arthritic rats on high-fat diet were similar to those found in normally fed arthritic rats, despite the fact that rats on high-fat diet had high leptin levels and low adiponectin levels before AA induction as we reported recently [17]. Production and secretion of leptin are primarily dependent on the amount of body fat mass [33]. Even though we observed a higher amount of mesenteric and epididymal fat in arthritic rats on high-fat diet than in normally fed arthritic rats, its secretion was less effective due to the inflammatory process. 

Under physiological conditions weight loss increases adiponectin levels [34, 35], and decreases visfatin levels [35, 36], but in inflammatory arthritis there were opposite results [19, 23–25, 37]. Our study has shown downregulation of adiponectin and upregulation of visfatin levels during AA to the same extent in all feeding regimes. Based on previously published reports [34, 38–42], the increased levels of corticosterone and proinflammatory cytokines may be involved in this regulation in arthritic rats. The worsening of arthritis in rats on high-fat diet is difficult to explain on the basis of the present results. The pathophysiology of obesity is very complex, and various pro-inflammatory factors can negatively contribute to the disease outcome. One of these factors could be the overfeeding-induced hyperleptinemia [17] which presumably took effect at the onset of AA, but not in the further progress of the disease.

### 4.2. AA Cachexia and mRNA Expressions in the ARC under Different Feeding Conditions

We have recently demonstrated an enhanced mRNA expression of IL-1*β* in the ARC of normally fed arthritic rats suggesting its role in arthritis-induced cachexia [19]. This study clearly shows that AA led to an up-regulation of IL-1*β* mRNA in the ARC, and moreover to reduced CART, unchanged POMC and increased NPY and AgRP mRNA expressions under all dietary interventions. In addition, food-restriction led to a more profound decrease of mRNA expressions for anorexigenic factors (POMC, CART, IL-1*β*) and marked increase of mRNA expressions for orexigenic factors (NPY, AgRP) compared to the other dietary regimes with or without arthritis.

The importance of IL-1*β* in the development of inflammatory cachexia was previously reported in experimental models of chronic inflammatory diseases, such as cancer or colitis, in which neutralization of IL-1*β* attenuated inflammatory anorexia [43, 44]. As for nutritional status, acute inflammation-associated anorexia in rats was reduced by prior food-restriction [45, 46]. Moreover, the sensitivity to IL-1*β*-induced anorexia was decreased in food-restricted rats [47, 48]. Therefore, we may hypothesize that down-regulation of IL-1*β* mRNA in the ARC observed in our study due to the food restriction may have contributed to the reduced cachectic response in chronic arthritis. It is difficult to assess a direct relationship between central IL-1*β* expression and inflammatory cachexia since we did not perfuse the rats in this study. However, regardless whether IL-1*β* mRNA expression in the ARC is of central origin, it may alter the secretion and function of key neuropeptides regulating energy balance. Noteworthy, previous studies demonstrated that IL-1*β* induced Fos immunoreactivity in both POMC and NPY-expressing neurons in the ARC [49] and *c-fos* mRNA expression in AgRP neurons in the ARC [50]. 

Similarly to our findings, in different models of acute and chronic inflammation-induced anorexia, increased mRNA expression of NPY and AgRP has been observed in the hypothalamus or ARC [50–54]. In the present study, mRNA levels of AgRP and NPY in the ARC were affected by both the energy status and the inflammation. Regarding the energy status, AgRP and NPY mRNAs were increased by food restriction as well as by overfeeding in healthy rats. One possible mechanism could lie in increased levels of corticosterone, a highly active stimulator of NPY/AgRP mRNA expressions [55]. Additionally, this finding reflects normal physiological response to fasting in food-restricted control rats [56, 57], but leptin resistance in control rats on high-fat diet as we described previously using the same feeding protocol [17]. As for the inflammation, AA was associated with low circulating leptin levels along with increased circulating ghrelin as well as corticosterone levels. These hormonal changes normally increase NPY/AgRP mRNA expression in the hypothalamus [55, 58, 59]. Therefore the upregulation of NPY/AgRP mRNA expression in the ARC is not surprising in arthritic rats, and reflects physiological response in cachexia. However, there is a discrepancy why upregulated expressions of orexigenic neuropeptides did not increase appetite in AA. It appears that inflammatory signaling in the hypothalamus has counteracted orexigenic response of NPY/AgRP neurons. This could involve negative interference with protein synthesis or release of neuropeptides, as demonstrated by Scarlett et al. [50]. 

POMC, a precursor of the anorexigenic *α*-melanocyte-stimulating hormone, and CART are colocalized in the same ARC neurons, and their mRNA expressions are stimulated by increased leptin signaling [12]. Consistently with low-leptin levels in AA, we found a decreased mRNA expression of CART in the ARC. This is in line with previous findings observed in cachectic tumor-bearing rats [53]. However, mRNA expression of POMC in the ARC did not differ significantly from controls in arthritic rats under all feeding conditions. Likewise, neither cachectic tumor-bearing rats [60, 61], nor LPS-treated rats [62] did demonstrate changes in hypothalamic or ARC mRNA expression of POMC. It thus appears that the anorexigenic contribution of the ARC POMC/CART mRNAs was less important during AA. 

Our results suggest that modulation of mRNA levels for the key hypothalamic neuropeptides regulating appetite is not the primary mechanism by which AA induces cachexia. On the other hand, increased IL-1*β* mRNA in the ARC in the present study highlights the importance of this pro-inflammatory cytokine among mechanisms through which chronic inflammation affects energy homeostasis. 

### 4.3. AA and Metabolic Response under Different Feeding Conditions

Chronic systemic inflammation in RA is implicated in the development of insulin resistance [63]. Furthermore, in inflammatory arthritis, overweight considerably contributes to insulin resistance, while calorie restriction together with disease-modifying antirheumatic drugs result in its improvement [64]. Our previous results showed downregulation of glucose transporter GLUT4 in adipocyte membranes in arthritic rats that may indicate decreased insulin sensitivity in AA [25]. In this study we did not observe any significant changes in HOMA index, in normally fed or arthritic rats on high-fat diet. Interestingly, arthritic rats on food restriction showed higher insulin sensitivity documented by a decreased HOMA index. Both adiponectin and visfatin are suggested to increase insulin sensitivity [10, 15], but regarding the similar plasma levels of adiponectin and visfatin in all arthritic groups, there cannot be assessed any link between these adipokines and improved insulin sensitivity in food-restricted arthritic rats. However, these rats showed markedly increased plasma ghrelin and suppressed plasma leptin levels compared to the other arthritic rats. Ghrelin has been found to stimulate insulin-induced glucose uptake in adipocytes [65]; and coadministration of acylated and unacylated ghrelin strongly improved insulin sensitivity in humans [66]. It thus appears that elevated ghrelin could be functionally associated with improved insulin sensitivity in food-restricted arthritic rats. On the other hand, a decrease of leptin levels associated with the reduction of body mass has been implicated in the pathogenesis of insulin-deficient diabetes, in which leptin replacement improved insulin sensitivity and decreased hepatic triglyceride content [67]. Moreover, leptin is proposed as an important antisteatotic hormone protecting nonadipose tissue from fat accumulation and lipotoxicity [68]. Here we found unchanged hepatic triglyceride concentration in food-restricted arthritic rats. However, hepatic cholesterol concentrations were increased in food-restricted arthritic rats compared to their healthy controls but not to normally fed arthritic rats. Thus, we cannot exclude that leptin downregulation may have detrimental consequences on lipid metabolism during chronic inflammation. 

Our results further show increased hepatic triglyceride and cholesterol levels in arthritic rats on high-fat diet compared to the arthritic rats on the other diets, despite normal values of HOMA index. An increased triglyceride deposition in the liver could be due to the excessive influx of free fatty acids from high-fat diet consumption as it was previously observed in rats [69] and humans [70]. In our study high-fat diet contained 45% calories as fat, predominantly as lard containing 39% saturated fat. Recent findings have shown a causal relationship between activation of inflammatory process and the concentrations of saturated free fatty acids in the diet [71]. Noteworthy, we observed increased IL-1*β* expression in the liver in arthritic rats on high-fat diet. Since IL-1*β* is at least partially involved in the development of hepatic steatosis [72], it may also play a considerable role in maintaining increased hepatic triglyceride concentration in arthritic rats on high-fat diet.

## 5. Conclusions

### 5.1. Arthritis Severity

In food restriction, the attenuation of chronic arthritis seems to be related to the downregulation of leptin and upregulation of ghrelin and corticosterone. In rats on high-fat diet the aggravation of AA is not related to the modulation of these hormones. However, initial hyperleptinemia at the time of the disease onset could have accelerated its progression. Circulating visfatin and adiponectin levels do not reflect the disease severity.

### 5.2. Control of Appetite

Overexpression of IL-1*β* in the ARC of arthritic rats on all dietary regimes indicates its role in AA- induced cachexia rather than anorexigenic neuropeptides CART and POMC. Overexpressions of orexigenic NPY and AgRP and lowered expressions of anorexigenic CART in the ARC suggest low effectivity of these compensatory mechanisms against negative energy balance during AA.

### 5.3. Metabolism

HOMA index in arthritic rats on high-fat diet, normal diet, and food-restriction does not indicate the development of insulin resistance. Elevated concentrations of total cholesterol and triglycerides in the liver of arthritic rats on high-fat diet indicate impaired lipid metabolism due to the increased inflammation in these rats.

## Figures and Tables

**Table 1:** Arthrogram score evaluation according to the degree of arthritic involvement for each paw.

Score	Degree of arthritic involvement
0	None
1	Erythema without swelling
2	Erythema + minor swelling (paw volume 1.5–1.7 ml)
3	Erythema + moderate swelling (paw volume 1.8–2.3 ml)
4	Erythema + severe swelling (paw volume 2.4–2.6 ml)
5	Erythema + the severest swelling (paw volume >2.6 ml)
